# Development of a Highly Sensitive Neurofilament Light Chain Assay on an Automated Immunoassay Platform

**DOI:** 10.3389/fneur.2022.935382

**Published:** 2022-07-25

**Authors:** Stephen Lee, Tatiana Plavina, Carol M. Singh, Kuangnan Xiong, Xiaolei Qiu, Richard A. Rudick, Peter A. Calabresi, Lauren Stevenson, Danielle Graham, Denitza Raitcheva, Christopher Green, Madeleine Matias, Arejas J. Uzgiris

**Affiliations:** ^1^Siemens Healthcare Laboratory, LLC, Berkeley, CA, United States; ^2^Biogen, Cambridge, MA, United States; ^3^Department of Neurology, Johns Hopkins University School of Medicine, Baltimore, MD, United States

**Keywords:** assay, neurofilament, biomarker, neurodegenerative article type: original research manuscript section, multiple sclerosis, amyotrophic lateral sclerosis

## Abstract

**Background:**

Neurofilament light chain (NfL) is an axonal cytoskeletal protein that is released into the extracellular space following neuronal or axonal injury associated with neurological conditions such as multiple sclerosis (MS), amyotrophic lateral sclerosis (ALS), and other diseases. NfL is detectable in the cerebrospinal fluid (CSF) and blood. Numerous studies on MS have demonstrated that NfL is correlated with disease activity, predicts disease progression, and is reduced by treatment with MS disease-modifying drugs, making NfL an attractive candidate to supplement existing clinical and imaging measures in MS. However, for NfL to achieve its potential as a clinically useful biomarker for clinical decision-making or drug development, a standardized, practical, and widely accessible assay is needed. Our objective was to develop a novel NfL assay on an automated, globally available immunoassay platform and validate its performance.

**Methods:**

A prototype NfL assay was first developed and evaluated on the ADVIA Centaur^®^ XP immunoassay system from Siemens Healthineers. The lower limit of quantitation (LLoQ), within-lab precision, assay range, cross-reactivity with neurofilament medium and heavy chains, and effect of interfering substances were determined. NfL assay values in serum and CSF were compared with radiological and clinical disease activity measures in patients with MS and ALS, respectively. This assay was further optimized to utilize serum, plasma, and CSF sample types on the Atellica^®^ IM system and transferred to Siemens' CLIA laboratory where it was analytically validated as a laboratory-developed test (LDT).

**Results:**

In this study, an LLoQ of 1.85 pg/mL, within-lab precision <6%, and an assay range of up to 646 pg/mL were demonstrated with the serum prototype assay. Cross-reactivity of <0.7% with the neurofilament medium and heavy chains was observed. Serum and CSF NfL assay values were associated with radiological and clinical disease activity measures in patients with MS and ALS, respectively. The optimized version of the NfL assay demonstrated specimen equivalence with additional plasma tube types and was analytically validated as an LDT.

**Conclusion:**

The analytical performance of the NfL assay fulfilled all acceptance criteria; therefore, we suggest that the assay is acceptable for use in both research and clinical practice settings to determine elevated NfL levels in patients.

## Introduction

One challenge for clinicians in managing neurodegenerative diseases is a lack of biomarkers that provide quantitative measures of underlying disease severity and activity for monitoring the effectiveness of disease-modifying therapies (DMT) (1–4). Molecular biomarkers that originate in the central nervous system (CNS), which is shielded by the blood-brain barrier, have been previously thought to be inaccessible to blood-based testing. With recent advances in diagnostic technology, measuring very low levels of such biomarkers is now possible using routine clinical laboratory platforms.

Neurofilament light chain (NfL) is a scaffolding protein found specifically in the neuronal cytoskeleton and is released into the extracellular space following axonal degeneration (5–7). As such, it is a promising biomarker that may have applications for stratifying disease severity, monitoring activity or progression of neurodegenerative disorders, and determining efficacy of treatments (8).

NfL levels are known to be correlated with the extent of axonal damage in a variety of neurological disorders (9). For multiple sclerosis (MS), it has been reported that baseline serum NfL (sNfL) is a predictor of long-term brain atrophy, development of new T2 lesions, T2 lesion volume, gadolinium (Gd+) lesions, and increased likelihood of progression from radiologically isolated syndromes or clinically isolated syndromes to clinically definite MS (10, 11). In addition, sNfL levels are higher and more variable in patients with evidence of active MS and decrease with a DMT (11). Multiple reports have shown that sNfL levels are responsive to treatment with MS DMTs (12–15). Similarly, NfL levels from the cerebrospinal fluid (CSF) of patients with amyotrophic lateral sclerosis (ALS) predict disease severity before it is clinically manifested (16).

It is thought that incorporation of NfL measurements into clinical decision-making may improve patient outcomes by allowing for earlier detection of neurodegenerative disease and by providing more effective monitoring to inform choice of appropriate therapeutic regimen and other care measures. Incorporation of NfL measurements into drug development may allow for informative enrollment into clinical trials and a sensitive measurement of treatment effect, thereby reducing required sample sizes for early-stage trials.

To incorporate NfL testing in clinical practice, measurement of NfL levels will need to be standardized and accessible. Most other assays used to generate evidence for the utility of NfL have run on research-use-only platforms (17). This report describes the development and validation of a novel NfL assay on a globally available clinical immunoassay platform. We present preliminary performance data from the prototype assay on the Centaur XP as well as validation data generated on the Atellica Solution platform after further optimization for use with universal sample types. This assay has been implemented in our CLIA laboratory as a laboratory developed test (LDT) and is being used in multiple clinical trials.

## Materials and Methods

### NfL Assay Development Overview

A summary of key development and optimization studies for the NfL assay is as follows: (1) screening and selection of capture and detection antibodies, (2) prototyping with capture and detection antibodies in reagents on an instrument, (3) preliminary feasibility studies, (4) optimization of assay parameters, (5) control system development, and (6) further development of additional sample types and technology transfer to the CLIA laboratory. In short, multiple antibody candidates were screened, and one antibody pair was selected for assay development. Antibodies were conjugated to biotin and an acridinium ester (tracer) for compatibility with Siemens immunoassay analyzers. Optimal assay formulations, critical assay parameters, and control systems were established prior to assessing the analytical performance. Using materials with HAMA, we were able to titrate our current heterophilic blocker to reduce heterophilic interference.

**Figure 1:**
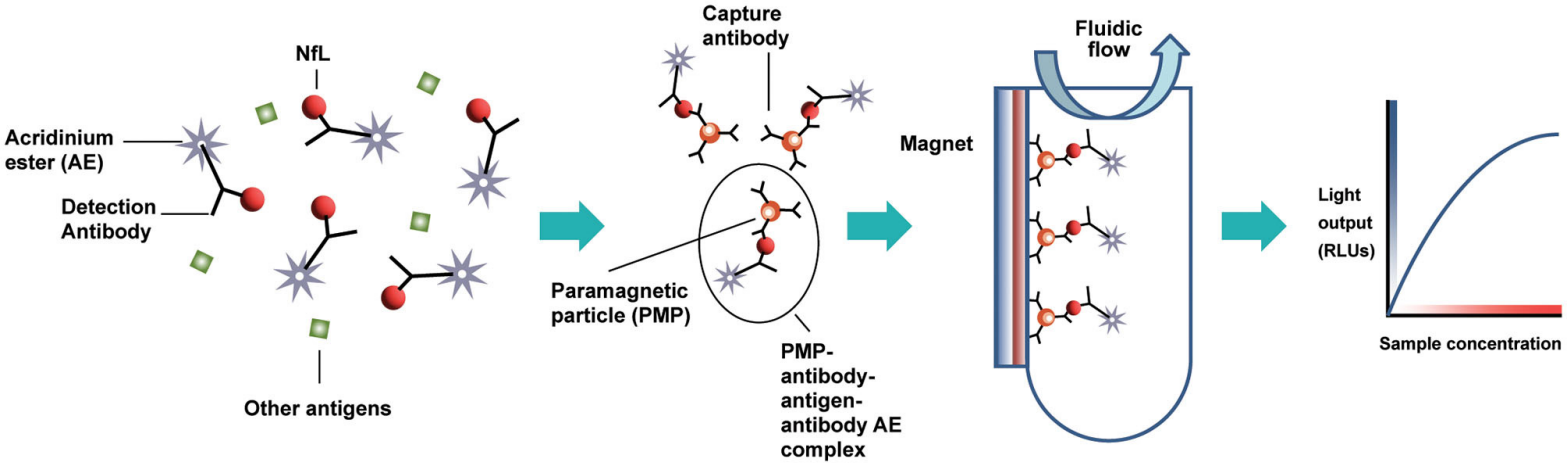
Acridinium ester-based automated immunoassay workflow, as implemented on the Siemens Centaur^®^ and Atellica^®^ testing platforms. AE, acridinium ester; PMP, paramagnetic particle; RLUs, relative light units.

The design of the NfL assay is immunometric. It uses solid-phase magnetic bead capture with one antibody and direct detection utilizing acridinium ester (AE) chemiluminometric detection with another antibody. The antibodies selected after screening were originally developed by Uman Diagnostics AB (Umea, Sweden), now a division of Quanterix Corporation (Billerica, MA). Accumulated light signal is related to NfL concentration in the sample. An initial serum NfL assay prototype was implemented and evaluated as a research assay on the ADVIA Centaur XP immunoassay system (Figure 1). Using the same materials formulated differently as reagents, a further universal sample-type (plasma/serum/CSF) NfL assay was optimized for and analytically validated on the Atellica Solution immunoassay system. Calibrators and control materials were also developed to enable reliable, highly sensitive, and quantitative reporting. Key differences between the assays are summarized in Table 1. The LDT NfL assay is only available as a testing service provided by Siemens Healthcare Laboratory.

**Table 1:** Summary of the neurofilament light chain (NfL) prototype and laboratory-developed test (LDT) assays' key differences.

	**Prototype**	LDT*
Instrument	ADVIA Centaur XP	Atellica Solution IM 1300
Assay throughput	Up to 75 tests per hour	Up to 171 tests per hour
Specimen types tested	Serum	Serum, plasma (K2 EDTA and lithium heparin), CSF

* *Available only as a test service through the Siemens Healthcare Laboratory*.

### Analytical Samples and Other Materials

Off-the-clot human serum pools (Access Biologicals, Vista, CA), K2 EDTA human plasma pools (Access Biologicals, Vista, CA), individual and matched sera (Access Biologicals, Vista, CA and BioIVT, Shirley, NY), K2 EDTA plasma (BioIVT, Shirley, NY), and lithium heparin plasma (BioIVT, Shirley, NY) were sourced. CSF samples were acquired from commercial sources (BioIVT, Shirley, NY). To establish the detection capabilities of the NfL assay at the low end of the assay range, contrived samples were utilized and prepared using NfL-depleted serum, plasma, or CSF. NfL-depleted matrixes were prepared by immuno-absorption. To prepare samples with higher NfL concentrations, recombinant human NfL and endogenous NfL from CSF were used as spikers.

Two tri-level serum-based quality control (QC) sets known herein as Siemens NfL QCs were prepared using off-the-clot human serum. One set known as endogenous QCs (EQC 1-3) consisted of one neat (or unspiked) serum pool and two other serum pools spiked with CSF to target NfL concentrations of 16 and 50 pg/mL. A second set known as recombinant QCs (RQC1-3) was prepared by spiking serum with recombinant human NfL to target NfL concentrations of 16, 50, and 450 pg/mL. The CSF sample used to spike endogenous QC materials originated from a donor with ALS. A tri-level plasma set known herein as Plasma Levels 1-3 consisted of one neat (or unspiked) K2 EDTA plasma pool and two other K2 EDTA plasma pools spiked to target NfL concentrations of 50 and 400 pg/mL with recombinant human NfL. Serum, plasma, and CSF samples sourced from individual donors were used for parallelism experiments. Parallelism samples were diluted serially with assay diluent, noted herein as NfL sample Diluent.

### Assay Precision

Repeatability and within-laboratory precision were assessed according to Clinical and Laboratory Standards Institute (CLSI) Document EP05-A3 (19) using a 20-day × 2 run × 2 replicate design with one reagent lot tested on one instrument. Aliquots of the six Siemens NfL QCs were prepared and frozen at −70°C prior to the start of the study. On the morning of each testing day, an aliquot was thawed to room temperature, mixed by inversion, and then transferred to a sample rack for duplicate testing. This process was repeated in a second run (at least 2 h after the first run) on the same testing day using a fresh aliquot. In total, each serum sample generated 80 measurements over 40 independent runs.

### Interfering Substances

Potential interferents such as intralipid (Sigma-Aldrich, St. Louis, MO), cholesterol (Lee Biosolutions, Maryland Heights, MO), human serum albumin (Lee Biosolutions, Maryland Heights, MO), human hemoglobin (Lee Biosolutions, Maryland Heights, MO), indirect Bilirubin (Conjugate; Lee Biosolutions, Maryland Heights, MO), direct Bilirubin (Lee Biosolutions, Maryland Heights, MO), rheumatoid factor serum (Lee Biosolutions, Maryland Heights, MO), and biotin (Sigma-Aldrich, St. Louis, MO) were spiked to minimum concentrations recommended by CLSI EP37 in three of the Siemens NfL QCs that spanned low, medium, and high levels of NfL (18). Control samples that did not contain an interferent were prepared by spiking the same samples with the storage buffer of each interferent. Interference was expressed as absolute percent bias between the mean test and control sample results.

### Specificity

Specificity was determined by spiking two other neurofilaments, neurofilament heavy chain (NfH) and neurofilament medium chain (NfM), into three Siemens NfL QCs and NfL-depleted serum. Purified bovine NfM and NfH (Origene, Rockville, MD) were each spiked into four samples spanning NfL assay range (0–500 pg/mL) at target concentrations of 1,000 pg/mL from 10 ng/mL stocks. Control samples were prepared by spiking the same four NfL samples with the storage buffer used to reconstitute the NfM and NfH. Cross-reactivity was calculated as the percent difference between the mean test and control sample results with respect to test analyte concentration.

### Sensitivity

Detection capability for limit of blank (LoB), limit of detection (LoD), and limit of quantitation was estimated in accordance with CLSI Document EP17-A2 and CLSI Document EP05-A3 (19, 20). Four different NfL-depleted human serum pools served as LoB samples and four NfL-depleted serum pools each spiked with neat pooled human serum at target concentrations of 1–4 times the LoB served as LoD samples. A human serum pool was diluted with NfL-depleted serum down to the LoB to yield 6 additional LLoQ panel samples. A human serum pool spiked with endogenous NfL from CSF to a target concentration of 16 pg/mL served as the highest LoQ sample. All LoB and LoD samples were assayed in replicates of five per run daily over 3 days with two reagent lots (60 total blank and 60 total low-level sample measurements per lot) in one instrument. LoB was calculated as the non-parametric 95th percentile of the rank-ordered results. LoD was determined parametrically from the low-level samples only using the equation LoD = LoB + CpSDL, where Cp is a multiplier to give 95th percentile of a normal distribution. LLoQ samples (target concentrations from assay LoB to 15-fold above the LoB) were tested two runs a day, four replicates per run for 5 days with two different reagent lots (40 total measurements per sample per lot) in one instrument. LLoQ was determined using the precision profile, where the LLoQ is calculated at the concentration corresponding to within-laboratory coefficient of variation of 20%.

### Linearity

Assay linearity was tested according to CLSI Document EP06-A (21) with three replicates of nine samples across the 0–500 pg/mL range. The nine serum samples of evenly spaced NfL concentration were prepared by mixing two contrived serum pools at opposite ends of the NfL assay range (**Figure 3A**). The highest contrived serum pool was spiked with recombinant NfL to slightly above the assay measurement range. The low concentration pool was an NfL-depleted serum pool. Linear regression analysis was performed between measured concentration and coded pool number.

### Serum Parallelism

Parallelism was defined as a condition in which dilution of test samples does not result in biased measurements of analyte concentration (with limits of 80–120% recovery). Parallelism was assessed using 10 serum samples from healthy individuals with relatively high NfL concentrations (range from 16 to 35 pg/mL). Each sample was diluted 1:2, 1:4, and 1:8. Measured concentrations of the diluted samples were multiplied by their dilution factor and compared to their neat concentration by percentage of recovery.

### Spike Recovery

The spike recovery of recombinant NfL in serum was performed with two serum cohorts. The first cohort consisted of five individual samples where one outlier was found. Therefore, a larger cohort of 50 individual donors was tested to determine interference frequency. To assess percent recovery, each sample was spiked with recombinant NfL, and individual and mean percentages of recovery were calculated and reported.

### Hook Effect

Hook (or prozone) effect was evaluated using a series of dilutions of a high NfL sample with two reagent lots. Recombinant NfL was spiked in serum to a target concentration of 500 ng/mL (1,000 times the upper limit of quantification [ULoQ]) as a high sample. The high sample and a series of dilutions of the high sample were tested with a control sample targeted at the ULoQ in replicates of three.

### Sample Stability

The stability of the serum samples was assessed at both ambient temperature and after freeze-thaws. Four individual donor serum samples were tested after storage on the bench at room temperature (20–25°C) for 4, 8, 24, and 48 h. Additionally, separate aliquots from the same four individual donors were also assessed for stability up to five freeze/thaw cycles.

### Method Comparison

Correlation of NfL results with Simoa^®^ NfL-light^®^ Advantage Kit (Quanterix, Billerica, MA) was examined using 458 clinical serum samples from the MSPATHS biorepository (*n* = 241) (22) and the ADVANCE clinical trial (*n* = 217) (11). Each sample was diluted 3-fold with Siemens NfL Sample Diluent and assayed in singlicate with one instrument. NfL values below LLoQ were excluded from the method comparison analysis. NfL results were back-calculated by dilution factor before method comparison analyses.

### Clinical Application

Serum samples and MRI images were collected from patients with MS enrolled in the ADVANCE study, a randomized, multicenter, double-blind, placebo-controlled study assessing the efficacy and safety of peginterferon beta-1a for patients with relapsing-remitting MS (11). NfL levels were measured using the assay described herein, and the number of new T2 lesions was derived from MRI images. CSF samples from healthy controls and patients with a diagnosis of clinically definite ALS were obtained from a research agreement between Biogen, Inc. (Cambridge, MA) and Iron Horse Diagnostics (Phoenix, AZ).

### Additional Studies (Only Performed With the LDT Version)

#### Specimen Equivalence

Specimen equivalence was assessed for three types of collection tubes: serum, K2 EDTA plasma, and lithium heparin plasma. Matched tube types from 40 individuals were tested without dilution.

#### Precision (K2 EDTA Plasma)

Repeatability and within-laboratory precision were assessed using a 5-day × two run × two replicate design with two reagent lots tested on one instrument. In short, aliquots of Plasma Levels 1–3 were prepared and frozen at −70°C prior to the start of the study. On the morning of each testing day, an aliquot was thawed to room temperature, mixed by inversion, and then transferred to a sample rack for duplicate testing. This process was repeated in a second run (at least 2 h after the first run) of the same testing day using a fresh aliquot. In total, each plasma sample had 20 measurements over 10 independent runs.

#### Plasma Parallelism

Parallelism was assessed for plasma tube types using 10 samples: five K2 EDTA and five lithium heparin samples from healthy individuals with relatively high normal NfL concentrations. Each sample was diluted 1:2, 1:4, 1:8, and 1:10. Measured concentrations of the diluted samples were multiplied by their dilution factor and compared to their neat concentration by percent recovery. Parallelism was demonstrated if recovery was within 80–120%.

#### CSF Parallelism

Seven individual CSF samples were serially diluted 10-, 20-, 40-, 80-, 160-, and 400-fold using NfL sample diluent. CSF was diluted with serum at least 10-fold to ensure that the test-matrix was primarily serum-based and compatible with the NfL assay. Relative recovery was calculated in comparison to a dilution-corrected concentration tested at 10-fold.

#### Onboard Sample Stability

Samples in the Atellica Solution are processed consecutively at a top speed of up to 440 samples per hour with continuous unattended loading for an entire workday. The sample management module scans and schedules processing of the samples, which are stored onboard at ambient temperature until each test order is processed. The samples are pipetted into individual reaction tubes that proceed independently through an incubator and pipetting stations with well-defined timings.

A sample stability study was performed to determine how long freshly thawed samples may remain in sample containers onboard the instrument and still provide reproducible results. Stability was assessed using the LDT with 3 test samples: one endogenous sample with low NfL concentration and two additional samples spiked with recombinant NfL to achieve medium and high NfL concentrations. Test samples were assayed at time 0 (baseline) and the 4-, 5-, 8-, and 9-h time points. The acceptance criterion was defined as ±20% of the baseline concentration.

## Results

### Analytical Performance (Serum Prototype Assay)

#### Precision

Repeatability and within-lab precision for Siemens Healthineers NfL QCs are summarized in Table 2. The within-laboratory percent coefficient of variation was <6% during the 20-day period.

**Table 2:** Precision with NfL prototype assay (serum).

		**Repeatability**		**Within-Lab precision**	
**Sample**	**Mean (pg/mL)**	**SD (pg/mL)**	**CV %**	**SD (pg/mL)**	**CV %**
Endogenous level 1 (EQC1)	7.65	0.26	3.4	0.39	5.1
Endogenous level 2 (EQC2)	17.23	0.57	3.3	0.76	4.4
Endogenous level 3 (EQC3)	54.18	0.96	1.8	1.66	3.1
Recombinant level 1 (RQC1)	17.89	0.39	2.2	0.58	3.2
Recombinant level 2 (RQC2)	54.95	0.84	1.5	1.37	2.5
Recombinant level 3 (RQC3)	438.03	7.49	1.7	12.17	2.8

*CV, coefficient of variation; NfL, neurofilament light chain; SD, standard deviation*.

#### Interference

Interferent concentration tested and absolute percent bias for the three Siemens NfL QCs are summarized in Table 3. Significant interference was considered absolute bias ≥10% for all NfL levels. Significant interference was observed with hemoglobin at a test concentration of 500 mg/dl (SI units 5 g/L). Bias for levels 1, 2, and 3 were 15, 16, and 22%, respectively. Lower concentrations of hemoglobin at 200 mg/dl showed no significant interference.

**Table 3:** Interference testing.

**Interferent**	**Substance test concentration convention Units (SI units)**	**% Bias in Level 1**	**% Bias in Level 2**	**% Bias in Level 3**
Intralipid	2,000 mg/dL(intentionally blank*)	9%	1%	2%
Cholesterol	500 mg/dL (12.95 mmol/L)	5%	3%	3%
Human serum albumin	6 g/dL (60 g/L)	9%	6%	8%
Human hemoglobin	500 mg/dL (5 g/L)	15%	16%	22%
	200 mg/dL (2 g/L)	1%	2%	2%
Direct bilirubin (conjugated)	60 mg/dL (712 μmol/L)	0%	5%	6%
Indirect bilirubin (unconjugated)	40 mg/dL (684 μmol/L)	4%	3%	2%
Rheumatoid factor serum	(193 U/mL)	0%	0%	8%
Biotin	3,500 ng/mL (14.3 μmol/L)	4%	2%	0%

*NfL, neurofilament light chain*.

* *Family of compounds that includes a wide variety of molecular weight substances, therefore marked intentionally blank*.

#### Specificity

Cross-reactivity of the Siemens NfL assay with purified NfM and NfH was below 0.7% for four different serum samples with NfL concentrations spanning the assay range (Table 4).

**Table 4:** Cross-reactivity assessment.

**Test substance**	**Concentration (pg/mL)**	**% Cross-reactivity**			
		**Target 0 pg/mL NfL-depleted serum**	**Target 7.65 pg/mL (Endogenous level 1)**	**Target 17.23 pg/mL (Endogenous level 2)**	**Target 438.03 pg/mL (Recombinant level 3)**
NfM	1,000	ND	0.0146%	0.0380%	0.3705%
NfH	1,000	ND	0.0233%	0.0011%	0.6780%

*ND was reported when the concentration difference between test and control samples is below the LoD. LoD, limit of detection; ND, not detectable; NfH, neurofilament heavy chain; NfM, neurofilament medium chain*.

#### Sensitivity

**Figure 2:**
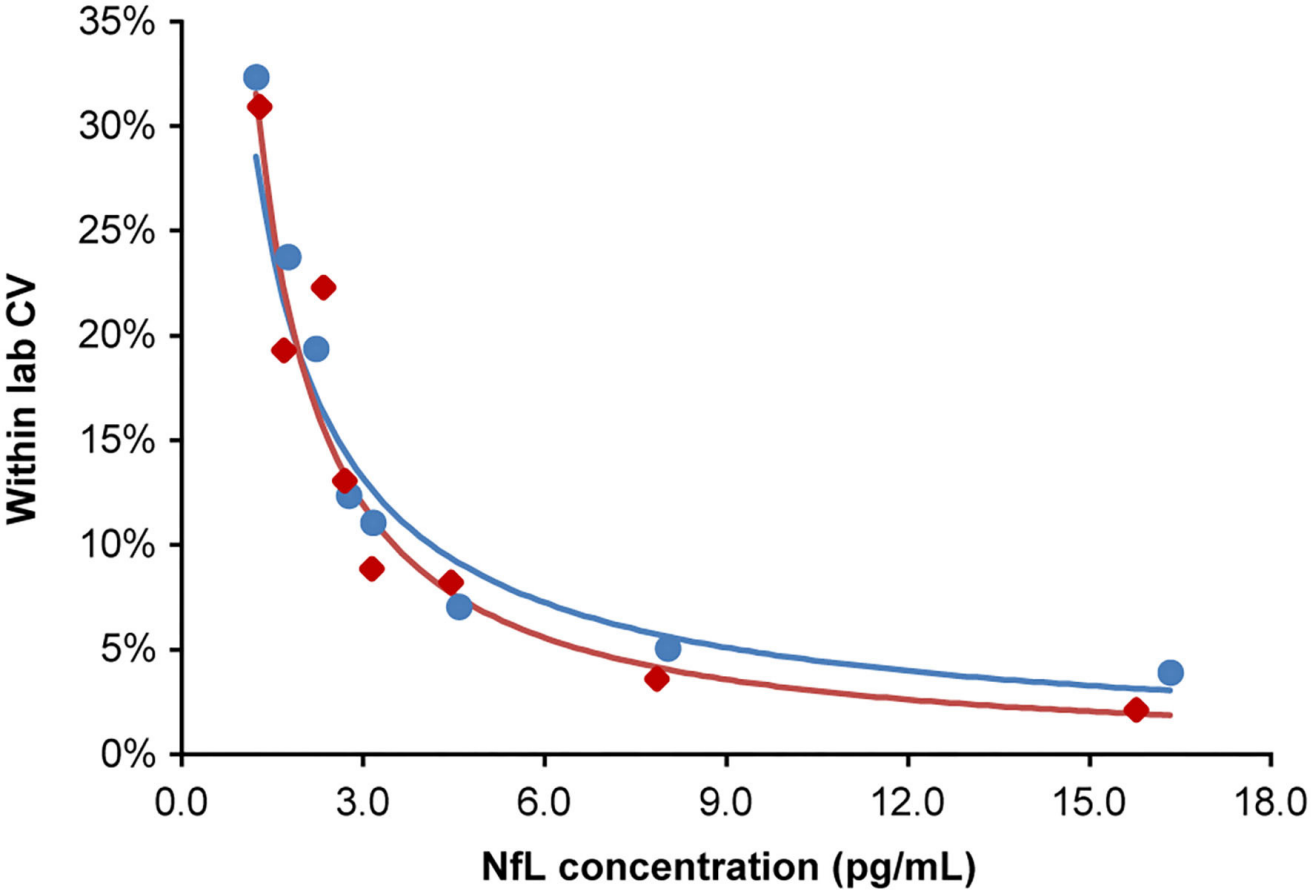
Lower limit of quantitation. Analytical samples at eight concentrations spanning the assay LoB (0.89 pg/mL) to 15-fold above the LoB. Samples generated from donor serum with endogenous NfL diluted in pooled donor serum first depleted of NfL by antibody immunoabsorption. Inter-run, inter-day, and inter-lot variations were tested (four replicates per sample; two runs per day over 5 days; two reagent lots). The lower limit of quantitation for lots 1 and 2 were 1.84 and 1.85 pg/mL, respectively, at a precision cut-off of 20% CV. The blue and red regression lines correspond to lots 1 and 2, respectively. CV, coefficient of variation; LoB, limit of blank; NfL, neurofilament light chain.

The highest LoB, LoD, and LLoQ results among the two reagent lots are reported for the assay. Three out of four LoD samples (45 of 60 measurements) were used to determine the LoD. One LoD sample after completion of the study was excluded, because the analyte concentration was too close to the LoB and could not be used for SD calculation. LoB was determined to be 0.89 pg/mL, and LoD was calculated as 1.49 pg/mL. LLoQ was determined using the precision profile method and equation of the power trendline fit and determined to be 1.85 pg/mL (Figure 2).

#### Linearity

**Figure 3:**
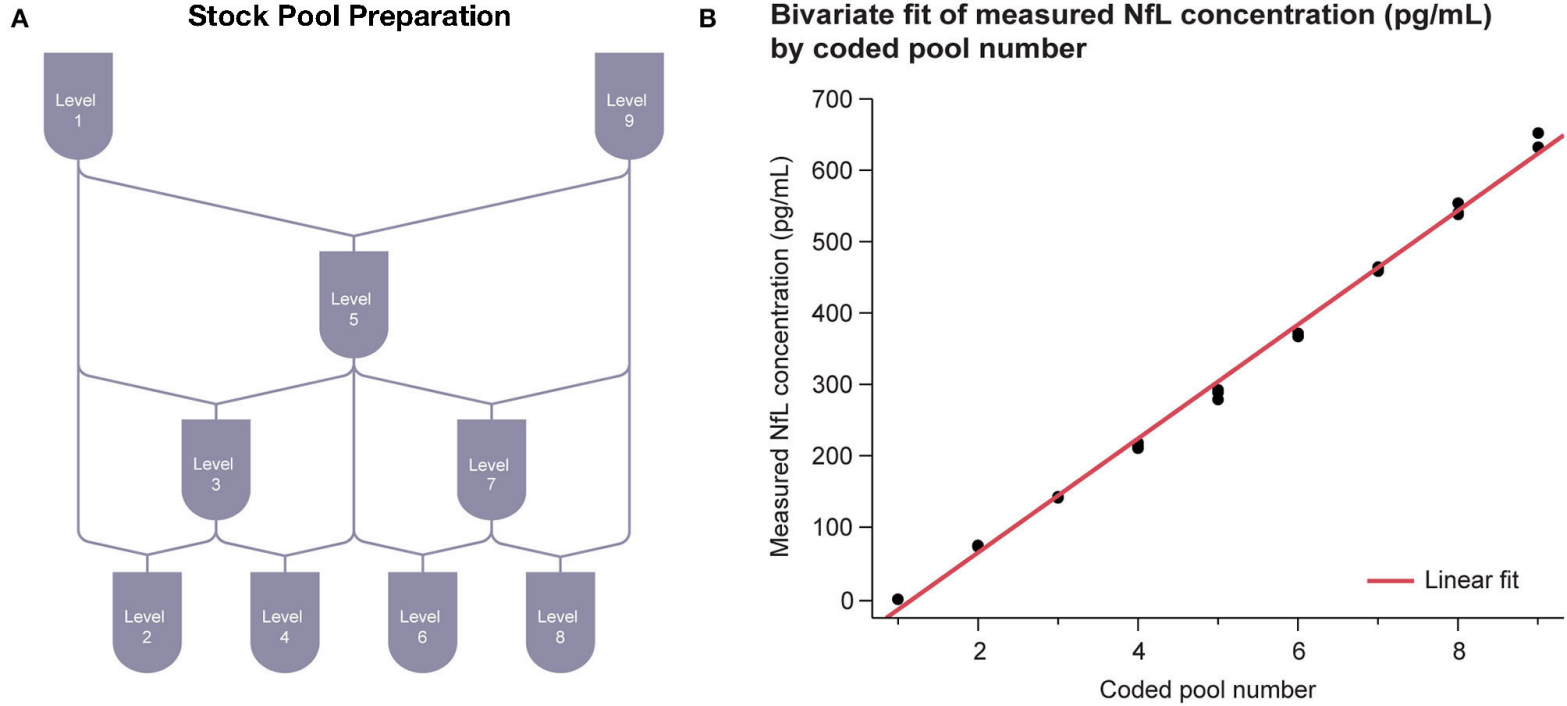
Serum linearity. **(A)** Sample stock pools with high (serum spiked with recombinant NfL to above ULoQ) and low (NfL-depleted serum below LLoQ) levels of NfL were used to prepare nine concentration levels evenly spaced across the assay range. **(B)** Regression between measured concentration and coded pool number was performed. LLoQ, lower limit of quantitation; NfL, neurofilament light chain; ULoQ, upper limit of quantitation.

Linearity of the NfL assay was observed across the range of 1–646 pg/mL. Linear regression results were *R*^2^ = 0.996 with *P* < 0.001 (Figure 3B).

#### Serum Parallelism

**Figure 4:**
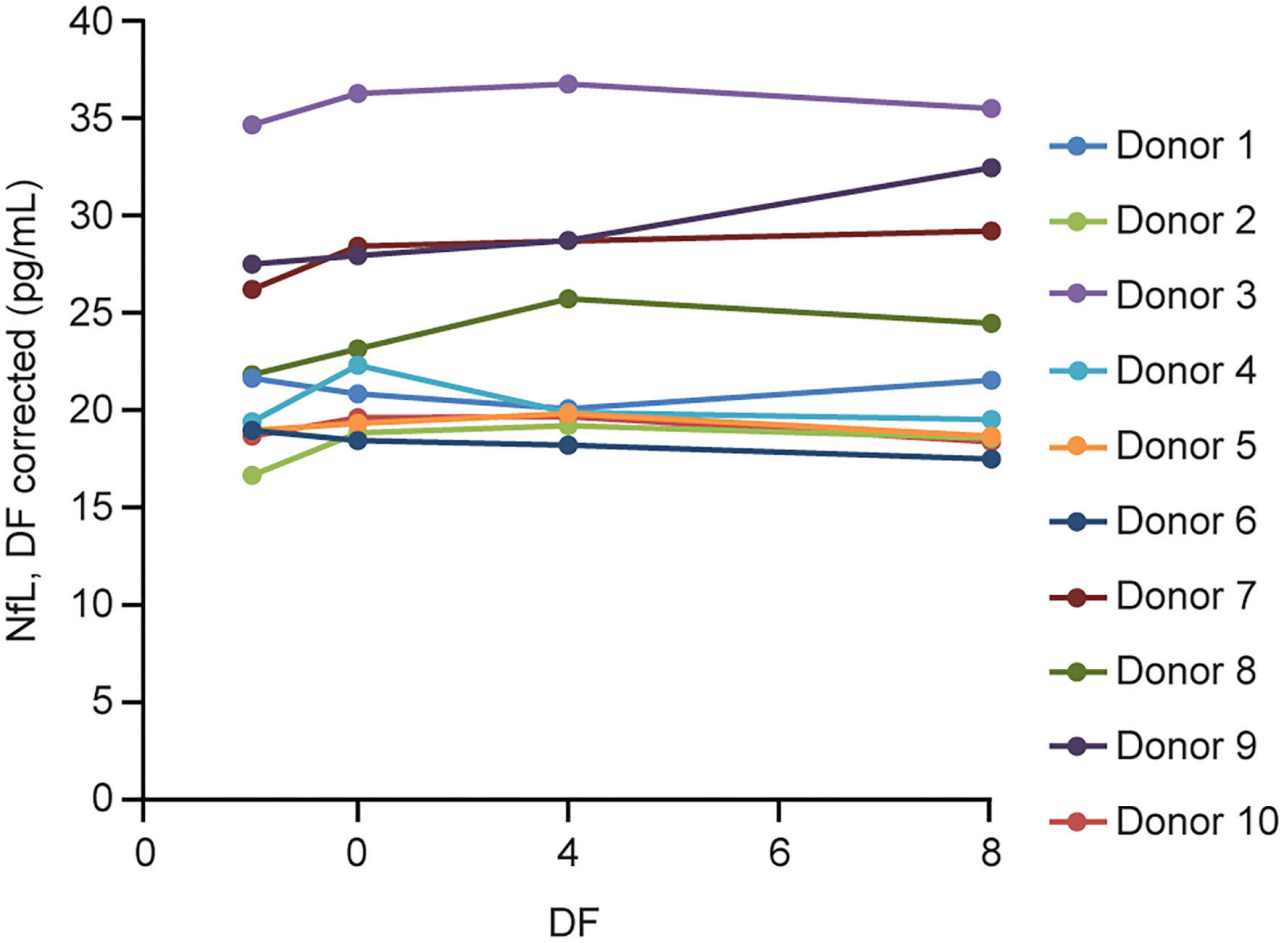
Serum parallelism. Ten individual serum samples with endogenous NfL levels >16 pg/mL (up to 35 pg/mL) were tested neat and serially diluted 2, 4, and 8 × using NfL sample diluent. DF, dilution factor; NfL, neurofilament light chain.

Parallelism was demonstrated with the prototype assay first in 10 individual sera with endogenous NfL levels ranging from 16 to 35 pg/mL. All the dilutions recovered within 80–120% of the neat measurement of each sample after adjusting for dilution factor (Figure 4).

#### Spike Recovery

Spike recovery was within our acceptance criteria of 80–120% for more than 95% of the samples (53 out of 55 of the samples, not shown).

#### Hook Effect

The hook or prozone effect is a phenomenon where formation of antibody-antigen immune complexes can be impaired when concentrations of the measurand (antigen or antibody depending on the type of assay) are very high. When there is a hook effect, there is a concentration point when the immunoassay measures less measurand when the measurand concentration is increasing, producing a hook shape on a graph of measurements. No hook effect was observed below 481 ng/mL for the two reagent lots tested (Supplementary Figure 2).

#### Sample Stability

Serum NfL stability was assessed at room temperature for up to 48 h and over five freeze-thaw cycles. All the samples were stable under these conditions as demonstrated by <5% difference from the control condition (Supplementary Figure 1).

#### Method Comparison With Quanterix Simoa Assay

**Figure 5:**
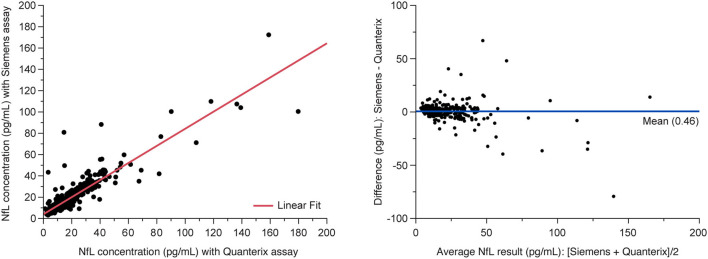
Method comparison between Siemens assay and Quanterix SIMOA assay. **(Left)** Deming regression and **(right)** Bland-Altman plots of agreement between methods. Data are from the MS PATHS and ADVANCE studies, selected over the range of Quanterix sNfL results and sample availability. sNfL, serum neurofilament light chain.

The analysis of MS patient serum samples (*n* = 418 above LLoQ) demonstrated a high correlation (*R*^2^ = 0.907) between NfL results from the Siemens ADVIA Centaur XP and the Quanterix Simoa platform (Figure 5).

### Analytical Performance (LDT Version of NfL Assay)

#### Specimen Equivalence

**Figure 6:**
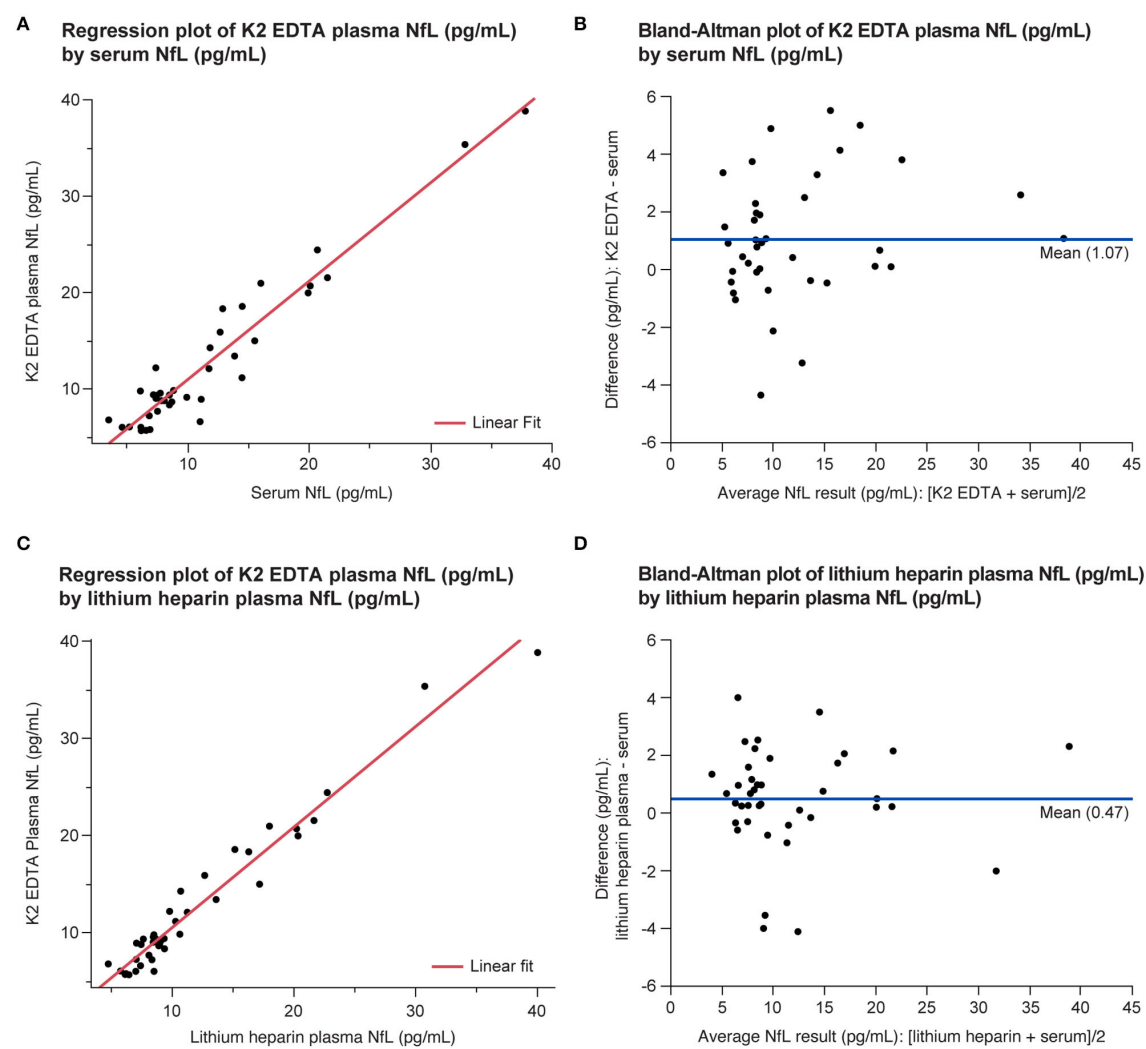
Equivalence of serum and plasma NfL levels in the LDT implementation of the NfL assay shown as regression **(A,C)** and Bland-Altman plots **(B,D)**. LDT, laboratory-developed test; NfL, neurofilament light chain.

All the three tube types demonstrated specimen equivalence (Figure 6). Linear fits for all the tube type combination comparisons were within the acceptance criteria of a slope equal to 1 ± 0.1 and y-intercept less than or equal to the LLoQ of the NfL assay (*P* < 0.0001).

#### Precision (K2 EDTA Plasma)

The repeatability and within-laboratory precision for the three tested plasma samples are summarized in Table 5. The within-laboratory percent coefficient of variation was ≤ 5.8% over the 5-day period for both reagent lots.

**Table 5:** Precision with the LDT version of the NfL assay (K2 EDTA plasma).

			**Repeatability**		**Within-lab precision**	
**Sample**	**Reagent lot**	**Mean (pg/mL)**	**SD (pg/mL)**	**CV%**	**SD (pg/mL)**	**CV%**
Plasma Level 1	Lot 1	10.1	0.3	2.8	0.4	3.9
Plasma Level 2		45.7	2.1	4.7	2.4	5.3
Plasma Level 3		346.3	16.4	4.7	19.8	5.7
Plasma Level 1	Lot 2	9.9	0.2	2.4	0.4	4.2
Plasma Level 2		46.0	2.4	5.2	2.7	5.8
Plasma Level 3		325.0	14.0	4.3	17.0	5.2

*CV, coefficient of variation; LDT, laboratory-developed test; NfL, neurofilament light chain; SD, standard deviation*.

#### Plasma Parallelism

**Figure 7:**
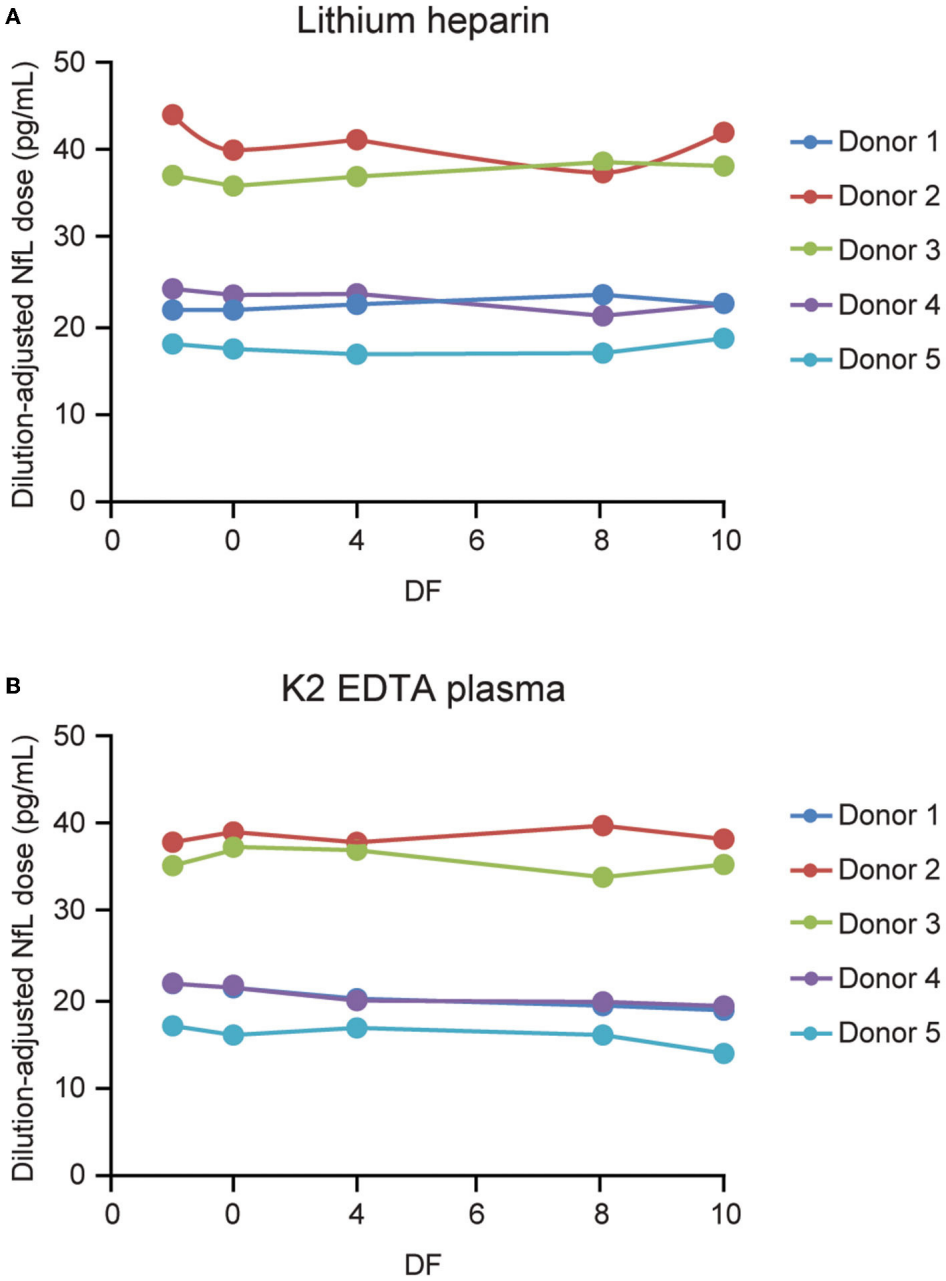
Parallelism for plasma tube types. Five matched lithium heparin **(A)** and K2 EDTA **(B)** samples with endogenous NfL levels of up to 45 pg/mL were tested, and diluted 2-, 4-, 8-, and 10-fold using NfL sample diluent. DF, dilution factor; NfL, neurofilament light chain.

Parallelism was demonstrated in matched K2 EDTA and lithium heparin plasma collected samples from 5 individuals (Figure 7). Endogenous levels ranged from 15.1 to 38.4 and from 16.7 to 38 pg/mL for the lithium heparin and K2 EDTA tube types, respectively. Percent recovery for 2-, 4-, 8-, and 10-fold dilutions with NfL Sample Diluent were all within 80–120% of the neat sample concentration.

#### CSF Parallelism

Seven CSF samples from normal individuals with NfL levels ranging from 206 to 1,439 pg/mL were tested serially diluted 10, 20, 40, 80, 160, and 400-fold using NfL Sample Diluent with the LDT version of the NfL assay. Parallelism was assessed using the 10-fold diluted measured concentration as the expected concentration instead of neat CSF because 3 of the 4 samples were out of the measurable assay range. All dilutions with measured concentrations above LLoQ for the 7 individual CSF donors tested exhibited 80–120% recovery in comparison to the 10-fold diluted concentration (not shown). Four of the seven CSF samples at starting concentrations <400 pg/mL did not have reportable results at 400-fold dilution.

#### Onboard Sample Stability

The mean, coefficient of variation, and percent recovery of all replicates (*n* = 5) per time point for each test sample are summarized in Supplementary Table 1. Recovery was 94.5–99.7% for all the testing time points when compared to baseline mean at time 0.

#### CLIA Validation

The optimized NfL assay for serum, K2 EDTA plasma, lithium heparin plasma, and CSF was transferred to the Siemens CLIA laboratory for validation. Results are summarized in Table 6.

**Table 6:** Summary of analytical validation of the LDT version of the NfL assay.

**Characteristic**	**Serum**	**Plasma**	**CSF**
Reportable range	3.9–500 pg/mL	4.9–477 pg/mL (*K2 EDTA)* 2.4–549 pg/mL (*Lithium Heparin*)	85.5–25,700 pg/mL
Reproducibility	4.9–8.4%	7.7–18.1% (*K2 EDTA)* 3.5–16.3% (*Lithium Heparin*)	4.0–16.5%
Method comparison	*Quanterix vs Atellica^®^ platform:* Avg Quantitation Difference: −8%; Pearson correlation: *R* = 0.995 *ADVIA Centaur XP vs. Atellica^®^ platform:* Average Quantitation Difference: 9%; Pearson correlation: *R* = 1.0	Tested for serum only; specimen equivalence was established based on precision and accuracy (CLSI EP35)	*Quanterix vs. Atellica^®^ platform:* Avg Quantitation Difference: −29%; Pearson correlation: *R* = 0.994 *ADVIA Centaur XP vs Atellica^®^ platform:* Average Quantitation Difference: −13.2%; Pearson correlation: *R* = 0.996
Specimen handling	NfL is stable under the following conditions: •Up to 6 freeze/thaw cycles •Up to 1 week at room temperature •Up to 2 weeks refrigerated •Frozen at −20°C for 1 year •Frozen at −80°C for 1 year	NfL is stable under the following conditions: •Up to 6 freeze/thaw cycles •Up to 1 week at room temperature •Up to 2 weeks refrigerated •Frozen at −20°C: 3 months (K2 EDTA) and 6 months (Lith Hep) •Frozen at −80°C: 6 months (K2 EDTA) and 1 year (Lith Hep)	NfL is stable under the following conditions: •Up to 6 freeze/thaw cycles •Up to 1 week at room temperature •Up to 1 week refrigerated •Frozen at −20°C for 3 months •Frozen at −80°C for 1 year
Interfering substances	Interference testing for endogenous substances. Assay interference was not observed in samples with the following substances and concentrations:•Hemoglobin below 500 mg/dL •Direct bilirubin below 60 mg/dL •Indirect bilirubin below 40 mg/dL •Albumin below 6 g/dL •Triglycerides below 2,000 mg/dL •RF below 193 U/mL •Biotin below 3,500 ng/mL •Neurofilament Heavy Chain below 1000 pg/mL •Neurofilament Medium Chain below 1000 pg/mL	Tested for serum only; specimen equivalence was established based on precision and accuracy (CLSI EP35)	Tested for serum only; specimen equivalence was established based on precision and accuracy (CLSI EP35)
Drug interference	Drug interference testing was performed using the following drugs used to treat patients with Alzheimer's and MS:•Donepezil •Rivastigmine •Memantine •Galantamine •Citalopram •Mirtazapine •Sertraline •Bupropion •Duloxetine •Imipramine •Ibuprofen •Siponimod •Acetaminophen •Aspirin •Beta interferon 1a •Beta interferon 1b •Fingolimod •Dimethyl fumarate •Teriflunomide •Ocrelizumab •Mitoxantrone •Caldribine •Alemtuzumab •Glucose •Drug interference (±20%) was observed in the presence of Mitoxantrone at concentrations >0.113 mg/dL.	Tested for serum only; specimen equivalence was established based on precision and accuracy (CLSI EP35)	Tested for serum only; specimen equivalence was established based on precision and accuracy (CLSI EP35)

*CLSI, clinical and laboratory standards institute; CSF, cerebrospinal fluid; MS, multiple sclerosis; NfL, neurofilament light chain*.

### Potential Clinical Application

**Figure 8:**
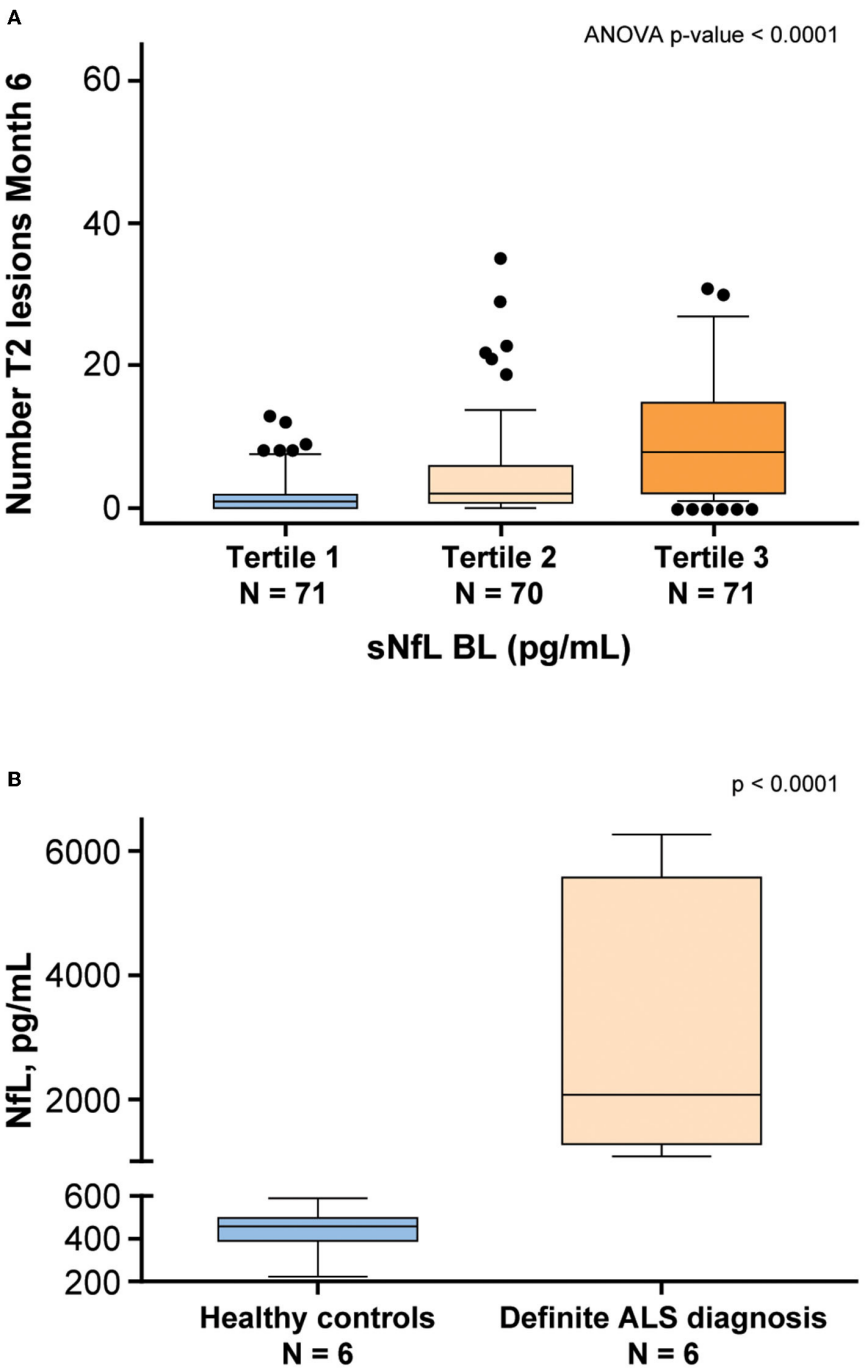
NfL levels are associated with neurodegenerative disease. **(A)** Patients with multiple sclerosis (MS) from the ADVANCE study (11) were separated into tertiles based on baseline (BL) sNfL. The vertical axis shows the number of T2 lesions that developed 6 months later. The lower and upper limits of each tertile were 5.6–11.3 pg/mL for tertile 1, 11.4–22.1 pg/mL for Tertile 2, and 22.4–100.4 pg/mL for tertile 3. **(B)** NfL levels were assessed in the CSF derived from healthy controls and patients with a definite ALS diagnosis. ALS, amyotrophic lateral sclerosis; ANOVA, analysis of variance; BL, baseline; CSF, cerebrospinal fluid; NfL, neurofilament light chain; sNfL, serum neurofilament light.

NfL levels in patients with neurodegenerative diseases were shown to be correlated with disease severity (9). To examine whether disease-specific trends in NfL using this assay, serum and CSF samples from patients with MS and ALS, respectively, were tested. Serum NfL data from patients with MS demonstrated an association with radiological disease activity (11). Baseline sNfL levels from 212 patients with MS were separated into tertiles and compared against the number of new T2 lesions that appeared 6 months later. The analysis of variance demonstrated that patients with higher NfL level exhibited a statistically significant (*P* < 0.0001) greater number of new T2 lesions after 6 months (Figure 8A). NfL levels were also assessed in the CSF derived from four healthy controls and four patients with a definite ALS diagnosis. Confirming previous studies (23, 24), NfL was significantly elevated (*P* < 0.0001) in the CSF of patients with ALS (Figure 8B). Additionally, NfL levels in the CSF were generally two orders of magnitude higher than the levels found in serum (23).

## Discussion

In this report, we describe the performance of a novel NfL assay that demonstrates operational and technical features that are compatible with Siemens automated AE-based immunoassay platforms that are used in laboratories for clinical trial testing applications. The assay provides a wide dynamic range and can be run on plasma (K2 EDTA and lithium heparin), serum, or CSF samples (Table 6). There is low cross-reactivity with the neurofilament medium and heavy chains, and the assay is not significantly affected by various interfering substances encountered in clinical specimens. Hemoglobin is a potential interferent. Healthy levels of hemoglobin are 14–17 g/dl for men and 12–15 g/dl for women (25). Generally, >100 mg/dl hemoglobin can have an effect on laboratory results. If a sample presents gross hemolysis, either it should be rejected based on previously established rejection criteria or hemoglobin should be quantified before an NfL test. The prevalence is expected to be low if phlebotomy and preanalytical factors are well-controlled. The LDT assay did not have significant interference when tested at 500 mg/dL (Table 6).

The NfL assay was designed for compatibility with widely available AE-based platforms. Instrument configurations are available for small-, medium-, and high-throughput laboratories. In our LDT validation, we utilized the Atellica Solution, which is the highest-throughput platform and the most recently launched hardware; this option would be appropriate for supporting largest global clinical trials and large clinical practices. Using this platform, the time to first results is 51 min, and throughput using a single immunoassay module on the Siemens Atellica solution is 171 samples per hour.

Many neurodegenerative diseases often progress stealthily with a long preclinical stage. It is during this prodromal stage that treatments could be most effective before serious, irreversible clinical symptoms become evident. In addition, MRI of the brain has shown that CNS atrophy occurs continuously in diseases such as MS even during periods of apparent clinical remission. Therefore, it appears that neurodegeneration may be clinically silent in younger patients who are having an ongoing low-level CNS injury but can compensate clinically because of reserve capacity and plasticity. However, the compensation eventually fails, and the ongoing process accelerates the time to future disability. Therefore, noninvasive biomarkers are needed that can detect underlying pathologies and monitor disease activity. Such biomarkers could also play a role in drug development by providing both a means to stratify patient populations and evidence that new drugs are reaching the appropriate molecular target. Furthermore, a biomarker of immune mediated neuronal injury could inform whether existing drugs are optimally effective and guide clinical decision-making for escalation of disease-modifying immunotherapies of various potencies. Currently, there is a lack of standardized, validated biomarkers in neurological diseases. NfL, however, is a very promising candidate, with evidence in the literature supporting the value of sNfL as a sensitive and clinically meaningful blood biomarker to monitor neuronal tissue damage and the effects of therapies on neurodegenerative disease (26–30). As we learn more about the strengths and limitations of NfL as a clinical biomarker, it is recognized that a highly sensitive, precise, and accurate test, accessible in the clinical practice setting, would be needed for widespread adoption of NfL in management of patients with neurodegenerative diseases.

Continuing use of this assay in clinical trials and biomarker validation studies, and with normative reference populations, is expected to help establish the utility of NfL in evidence-based decision-making in care of patients with MS and as a potential measure of neurodegeneration, which may accelerate development of treatments that slow disease progression in other diseases such as ALS. Demonstration of the performance of an NfL assay on a routine clinical laboratory platform is an important step toward bringing NfL into clinical practice and developing drugs for a wide range of potential applications in neurology.

## Data Availability Statement

The raw data supporting the conclusions of this article will be made available by the authors, without undue reservation.

## Ethics Statement

The following Ethics Committees and Institutional Review Boards of the participating institutions in Multiple Sclerosis Partners Advancing Technology and Health Solutions (MS PATHS), funded by Biogen, gave ethical approval for this work. 1. University of Rochester Research Subjects Review Board. 2. New York University School of Medicine Institutional Review Board. 3. Washington University in St. Louis Institutional Review Board. 4. Western Institutional Review Board. 5. Cleveland Clinic Institutional Review Board, Cleveland Clinic Institutional Review Board. 6. Johns Hopkins Medicine Institutional Review Board. 7. Ethik-Kommission der Ärztekammer Westfalen-Lippe und der Westfälischen Wilhelms-Universität Münster. 8. Comité Ético de Investigación Clínica del Hospital Universitario Vall d'Hebron. 9. Ethik-Kommission des Fachbereichs Humanedizin der Philipps-Universität Marburg. 10. Ethikkommission an der Technische Universität Dresden. The following Ethics Committees and Institutional Review Boards of the participating institutions in Pegylated interferon β-1a for relapsing-remitting multiple sclerosis (ADVANCE), funded by Biogen, gave ethical approval for this work. 1. Comité d'Ethique UCL Saint-Luc, Avenue Hippocrate 55.14, Tour Havey, niveau 0, Dr. J. M. Maloteaux (Président)/Prof. M. F. van den Hove (Secrétaire), Brussels 1200, Belgium. 2. Regionaal Ziekenhuis Sint-Trudo VZW - Ethisch comité, Diestersteenweg 100, Campus Sint-Jozef, Sint-Truiden, 3800, Belgium. 3. Ethics Committee for Multi-centre Trials, 5 Sveta Nedelya Sq, Sofia, 1000, Bulgaria. 4. University of Western Ontario Health Sciences Research Ethics Board, 1393 Western Road, Room 4180, London, Ontario, N6G 1G9, Canada. 5. Comite d'Ethique-Hopital Saint Luc-Edifice Cooper, 3981 Boulevard St. Laurent, Mezzanine 2-Bureau M-207, Montreal, Quebec, Canada. 6. Comite de Evaluacion Etico Cientifico del Servicio de Salud Metropolitano Sur Oriente, Avenida Concha y Toro 3459, Oficina de Investigaciones Medicas, Block Central, Puente Alto, Santiago 8207257, Chile. 7. EC of Fundacion del Caribe para la Investigacion Biomedica (Fundacion BIOS), Cra 44 No 72−131 Oficina 201, Barranquilla, Colombia. 8. Comité de Ética e Investigaciones de la Fundación Clínica Abood Shaio, Diagonal 115A Numero 70C-75, Bogota, Colombia. 9. Central Ethics Committee, Ksaver 200A, Zagreb, 10000, Croatia. 10. Local Drug Committee at Clinical Hospital Sestre Milosrdnice, 29 Vinogradska Street, Zagreb, 10000, Croatia. 11. Local Drug Committee at Clinical Hospital Dubrava, 6 Gojka Suska Avenue, Zagreb, 10000, Croatia. 12. Drug Committee Osijek, 4 J.Huttler Street, Osijek, 31000, Croatia. 13. Eticka komise Fakultni nemocnice Ostrava, 17. listopadu 1790, Ostrava 708 52, Czech Republic. 14. Eticka komise Krajske zdravotni, a.s. - Nemocnice Teplice, o.z., Duchcovska 53, Teplice 415 29, Czech Republic. 15. Eticka komise Fakultni nemocnice Olomouc a LF UP v Olomouci, I. P. Pavlova 6, Olomouc 775 20, Czech Republic. 16. Eticka komise Fakultni nemocnice v Motole, V Uvalu 84, Praha 5, 150 06, Czech Republic. 17. Eticka komise Fakultni nemocnice Brno, Jihlavska 20, Brno 625 00, Czech Republic. 18. Eticka komise Vseobecne fakultni nemocnice v Praze, Na Bojisti 1, Praha 2, 128 08, Czech Republic. 19. Eticka komise Fakultni nemocnice Brno, Jihlavska 20, Brno 625 00, Czech Republic. 20. Tallinn Medical Research Ethics Committee, Hiiu 42, National Institute for Health Development, Room 124, Tallinn EE-11619, Estonia. 21. CPP Sud-Méditerranée II - Hôpital Salvator, 249 Boulevard de Sainte Marguerite, Marseille 13009, France. 22. Independent Ethics Committee at Ltd S. Khechinashvili University Clinic, 33, Chavchavadze Ave, Tbilisi 179, Georgia. 23. Independent Ethics Committee at Petre Sarajishvili Institute of Neurology, 13, Tevdore Mgvdeli street, Floor 3, Tbilisi 112, Georgia. 24. Independent Ethics Committee of Ltd Research Institute at Clinical Medicine, 13, Tevdore Mgvdeli street, Tbilisi 112, Georgia. 25. Independent Ethics Committee at Ltd Samkurnalo Combinati, 16, Kavtradaze street, Tbilisi 186, Georgia. 26. Ethikkommission an der medizinischen Fakultät der Heinrich-Heine-Universität, Moorenstraße 5, Düsseldorf 40225, Germany. 27. Ethik-Kommission der Ärztekammer Westfalen-Lippe und der Medizinischen Fakultät der WWU Munster, Von-Esmarch-Straße 62, Medizinische Fakultät der Westfälischen Wilhelms-Universität Münster, Münster 48149, Germany. 28. Ethik-Kommission der Bayerischen Landesärztekammer, Mühlbaurstraße 16, München 81677, Germany. 29. Ethik-Kommission bei der Ärztekammer Niedersachsen, Berliner Allee 20, Hannover 30175, Germany. 30. Landesamt für Gesundheit und Soziales Berlin, Fehrbelliner Platz 1, Geschäftsstelle der Ethikkommission des Landes Berlin, Berlin 10707, Germany. 31. Ethikkommission des FB Medizin der Philipps-Universität Marburg, Baldingerstraße, Marburg 35032, Germany. 32. Ethik-Kommission an der Medizinischen Fakultät der Universität Leipzig, Härtelstraße 16–18, Institut für Klinische Pharmakologie, Leipzig 40107, Germany. 33. Ethik-Kommission der Ärztekammer Hamburg, Humboldtstraße 67a, Hamburg 22083, Germany. 34. Ethik-Kommission der Medizinischen Fakultät Friedrich-Alexander-Universität Erlangen-Nürnberg, Krankenhausstraße 12, EG, Raum 106, Erlangen 91054, Germany. 35. Ethik-Kommission der Medizinischen Hochschule Hannover, Carl-Neuberg-Straße 1, Hannover 30625, Germany. 36. Ethik-Kommission der Bayerischen Landesärztekammer, Mühlbaurstraße 16, München 81677, Germany. 37. Landesamt für Gesundheit und Soziales Berlin, Fehrbelliner Platz 1, Geschäftsstelle der Ethikkommission des Landes Berlin, Berlin 10707, Germany. 38. Ethikkommission der Landesärztekammer Hessen, Im Vogelsgesang 3, Frankfurt 60488, Germany. 39. Ethik-Kommission bei der Landesärztekammer Baden-Württemberg, Jahnstraße 40, Stuttgart 70597, Germany. 40. Ethikkommission der Medizinischen Fakultät der Ruhr-Universität Bochum, Bürkle-de-la-Camp-Platz 1, BG-Kliniken Bergmannsheil, Bochum 44789, Germany. 41. Ethikkommission der Ärztekammer Nordrhein, Tersteegenstraße 9, Düsseldorf 40474, Germany. 42. Ethik-Kommission der Medizinischen Fakultät der Ludwig-Maximilians Universität, München, Pettenkoferstr. 8a, München 81675, Germany. 43. National Ethics Committee for Clinical Trials, Mesogeion 284, Athens 15562, Greece. 44. Institutional Ethics Committee - Deenanath Mangeshkar Hospital, Karve Road, Deenanath Mangeshkar Hospital and Research Centre, 30C, Erandwane, Maharashtra, Pune 411004, India. 45. Ethics Committee Poona Hospital Research Centre, 27 Sadashiv Peth, Maharashtra, Pune 411030, India. 46. All India Institute of Medical Sciences, 29 Aurobindo Marg, Ansari Nagar, New Delhi 110029, India. 47. MCRI Professional Ethics Committee for Research on Human Subjects, Opposite Mahamarg Bus Stand, Mumbai Naka, Curie Manavata Center, Suyojit City Centre, Maharashtra, Nashik 422004, India. 48. KMCH Ethics Committee, Avanashi Road, Tamil Nadu, Coimbatore 641014, India 49. Ethics Committee, Jaslok Hospital and Research Centre, 15, Dr. G. Deshmukh Marg, Pedder Road, Maharashtra, Mumbai 400026, India 50. Ethics Committee, Rajinder Nagar, Sir Ganga Ram Hospital, New Delhi 110060, India. 51. Ethics Committee Vidyasagar Institute of Mental Health and Neuro-Sciences, 1, Institutional Area, New Delhi, Nehru Nagar 110065, India. 52. Institutional Ethics Committee Manipal Hospital and Manipal Heart Foundation, Airport Road, Department of Vascular Surgery, 98, Rustom Bagh Road, Karnataka, Bangalore 560017, India. 53. Central India Medical Research Ethics Committee, Dr.S.M.Patil's Hospital, 2nd Floor, Yugadharma Complex, Ramdaspeth, Nagpur 440010, India. 54. Independent Ethics Committee, TN Medical College and BYL Nair Ch. Hospital, Dept of Clinical Pharmacology, Old RMO Bldg, Mumbai 400008, India. 55. Max Healthcare Ethics Committee, 1, Press Enclave Road, Saket 110017, India. 56. Independent Ethics Committee, Cerebrovascular and Vasculities Research Foundation, Flat “B” Balaji Villa, 9/2, Rajarathinam Street, Kalipauk 600 010, India. 57. G.K. Hospital Ethics Committee for Human Subject Research, 11/2 Old Palasia, Department of Neurology, Indore 452018, India. 58. Central Ethical Committee, Medical Sciences Complex, Nitte University, Mangalore 575018, India. 59. Ethics Committee - SMS Medical College and attached Hospital, Jawaharlal Nehru Marg, Jaipur 302004, India. 60. Well Care Research Ethics Committee, 25 New Jagnath Road, Behind A.G Office, Gujarat, Rajkot 360001, India. 61. Sujlam Independent Ethics Committee, 2nd, floor, AMA House, Near Natraj Cinema, Ashram Road, Ahmedabad 380009, India. 62. The Ethics Committee Of Sri Aurobindo Seva Kendra, Sri Aurobindo Seva Kendra, 1H, Gariahat road (South), Kolkata 700068, India. 63. SAHEB Central Ethics Committee, Amritsar, 1st floor, 143-144/7, Near Gurunanak Bhawan, City Centre Market, Amritsar 143001, India. 64. Bangalore Central Ethics Committee, No. 1423, Kullappa Circle, Kullappa Layout, St. Thomas Town, Kammanahalli, Bangalore 560084, India. 65. The Ethics Committee for Clinical Trials on Medicinal Products, Aizkraukles Street 21-113, Riga LV-1006, Latvia. 66. Comite de Etica e Investigacion del Instituto Biomedico de Investigacion A.C., Sierra Fria 218, Fraccionamiento Bosques del Prado Norte, Aguascalientes 20217, Mexico. 67. Comite de Bioetica del Instituto de Ciencias Biomedicas Angeles, Camino a Santa Teresa 1055, Torre Especialidades, Colonia Heroes de Padierna, Mexico City, DF 10700, Mexico. 68. Comite Bioetico para la Investigacion Clinica S.C. Institutional Review Board, Puebla 422 despacho 4, Col. Roma Sur, Mexico, DF 6700, Mexico. 69. Comision de Ética del Hospital San José Tec de Monterrey y de la División de Ciencias de la Salud, Av. I. Morones Prieto 3000 Pte, Despacho 1, Col. Los Doctores, Monterrey, Nuevo Leon 64170, Mexico. 70. Tijuana General Hospital, Comite de Ensenanza e Investigacion, Aveinda Centenario Numero 10851 Zona Rio, Instituto de Servicios de Salud Publica Del Estado de Baja California, Tijuana, Baja California 22320, Mexico. 71. Comité de Ética del Hospital General de Tijuana, Av. Centenario #10851, Zona Rio, C.P. 22320, Tijuana, B.C., Mexico. 72. Comité de Ética e Investigación Christus Muguerza del Parque SA de CV, Calle Dr. Pedro Leal Rodriguez 1802, Colonia Centro, Chihuahua 31000, Mexico. 73. AZM METC, Oxfordlaan 10, Kamer 4.R1.33, Maastricht 6202 AZ, Netherlands. 74. Multi-Region Ethics Committee, PO Box 5013, Level 2, 1-3 The Terrace, Wellington, New Zealand. 75. Comite de Etica para la Investigacion de la Universidad de San Martin de Porres Clinica CADAMUJER, Avenida Alameda del Corregidor 1531, Urbanizacion Los Sirius, Las Viñas, La Molina, Lima 12, Peru. 76. Comité de Ética en Investigación Biomédica del Hospital Nacional Dos de Mayo, Parque Historia de La Medicina Peruana, s/n, Av. Grau, Cuadra 13, Lima, Lima 01, Peru. 77. Komisja Bioetyki Uniwersytetu Medycznego w Lodzi, Kosciuszki 4, Lodz 90-419, Poland. 78. National Ethics Committee for Clinical Trial on Medicine, 48 Aviator Sanatescu Street, Sector 1, Bucharest 11478, Romania. 79. Ethics Committee at the Federal Service on Surveillance in Healthcare and Social Development of RF, Petrovskiy Bulvar, 8, stroenie 2, Moscow 127051, Russia. 80. Ethics Committee at Siberian Regional Medical Centre, Ulitsa Kainskaya, 13, Novosibirsk 630007, Russia. 81. Ethics Committee within Chelyabinsk City Clinical Hospital #3, Prospect Pobedy, 287, Chelyabinsk 454136, Russia. 82. Ethics Committee at Republican Clinical Hospital for Rehabilitation Treatment, Ulitsa Vatutina, 13, Kazan 420021, Russia. 83. Ethics Committee within Bashkiria State Medical University, Ulitsa Lenina, 3, Ufa 450000, Russia. 84. Ethics Committee within Smolensk Regional Clinical Hospital, Prospect Gagarina, 27, Smolensk 214018, Russia. 85. Ethics Committee at City Clinical Hospital # 11, Ulitsa Dvintsev, 6, Moscow 127018, Russia. 86. Ethics Committee within Central Clinical Hospital #2 n.a. N.A. Semashko OAO RZhD, Ulitsa Budayskaya, 2, Moscow 129128, Russia. 87. Ethics Committee within Siberian State Medical University, Moscovskiy Tract, 2, Tomsk 634050, Russia. 88. Ethics Committee within Moscow Medical Academy n.a. I.M. Sechenov, 8 Ulitsa Trubetskaya stroenie 2, City Clinical Hospital #61, Moscow 119992, Russia. 89. Ethics Committee at City Hospital #2 – Kransodar Multispeciality Treatment and Diagnostics Unit, Ulitsa Krasnykh Partizan, 6, korp. 2, Krasnodar 350012, Russia. 90. Ethics Committee at Perm State Medical Academy, Ulitsa Kuybysheva, 39, Department of General Surgery, Perm 614990, Russia. 91. Ethics Committee at Perm State Medical Academy, Ulitsa Kuybysheva, 39, Department of General Surgery, Perm 614990, Russia. 92. Ethics Committee within Research Institute of Neurology of RAMS, Volokolamskoye Shosse, 80, Moscow 125367, Russia. 93. Local Ethics Comittee at Clinical Center NIS, 48 Zorana Djindjica Boulevard, Nis 18000, Serbia. 94. Local Ethics Committee at Military Medical Academy, 17 Crnotravska Street, Belgrade 11000, Serbia. 95. Local Ethics Comittee at Clinical Center of Serbia, 2 Pasterova Street, Belgrade 11000, Serbia. 96. Local Ethics Comittee at Clinical Hospital Center Kragujevac, 30 Zmaj Jovina Street, Kragujevac 34000, Serbia. 97. CEIC Hospital La Paz, Paseo de la Castellana, 261, Hospital General - Comité Ético de Investigación Clínica, Planta 8, Madrid 28046, Spain. 98. CEIC de Andalucía, Avenida de la Innovación s/n, Servicio de Investigación y Desarrollo Personal, Edificio Arena 1, Consejería de Salud - Dirección General de Procesos y Formación, Sevilla 41020, Spain. 99. Centro de Farmacovigilancia de Andalucia, Avenida Manuel Siurot, s/n, Servicio de Farmacologia Clinica, Edificio de Laboratorios - 1ª Planta, Hospitales Universitarios Virgen del Rocio, Sevilla 41013, Spain. 100. CEIC Hospital Virgen Macarena, Calle Dr. Fedriani, 3, Comité de Ensayos Clínicos, Planta 2, Sevilla 41009, Spain. 101. CEIC Hospital Universitario Reina Sofía, Avenida Menéndez Pidal, s/n, Planta 1, Edificio de Consultas Externas, Córdoba 14004, Spain. 102. Instituto de Investigación Hospital 12 de Octubre (i+12), Avenida de Córdoba s/n, Area de Gestión de Proyectos - Unidad Administrativa CEIC, Planta 6ª, Centro de Actividades Ambulatorias - Bloque D, Madrid 28041, Spain. 103. Central Commission on Ethics Questions of the MoH of Ukraine, Vulytsya Narodnogo Opolchennya, 5, Kyiv 3680, Ukraine. 104. Committee on Ethic Questions of Kyiv City Clinical Hospital #4, Vulytsya Solomyanska, 17, Kyiv 3110, Ukraine. 105. Commission on Ethics Questions of Municipal Institution of Healthcare Kyiv Regional Clinical Hospital, Vulytsya Baggovutivska, 1, Thoraco-Pulmonary Centre, Kyiv 4107, Ukraine. 106. Commission on Biomedical Ethics Questions of Chernivtsi Regional Psychiatric Hospital, Vulytsya Musorgskogo, 2, Chernivtsi 58018, Ukraine. 107. Commission on Ethics Questions of Crimean Republican Institution Clinical Hospital n.a N.A. Semashko, Vulytsya Kyivska, 69, Simferopol 96017, Ukraine 108. Bioethics Committee within Dnipropetrovsk State Medical Academy, Dzerzhinskogo vulytsya, 9, Dnipropetrovsk 49044, Ukraine 109. Ethics Commission of Ukrainian State Research Institute of Medical and Social Problems of Disability, Provulok Radyanskyy, 1a, Dnipropetrovsk 49027, Ukraine 110. Committee on Medical Ethics of Central Clinical Hospital of Railways of Ukraine, Provulok Balakireva, 5, Kharkiv 61103, Ukraine 111. Commission on Bioethic Questions of Donetsk National Medical University named after M. Horkyy, Prospekt Illicha, 16, Donetsk 83003, Ukraine 112. Comission on Ethics Questions of Vinnytsa Regional Psychoneurological Hospital n.a. Yuschenko, Vulytsya Pyrogova, 109, Vinnytsya 21005, Ukraine 113. Commission on Ethics Questions of Odesa Regional Clinical Hospital, Vulytsya Zabolotnogo, 26, Odesa 65025, Ukraine 114. Commission on Ethics Questions of Poltava Regional Clinical Hospital n.a. M.V. Sklifosovskyy, Vulytsya Shevchenka, 23, Poltava 36024, Ukraine 115. Local Ethic Commission of Institute of Neurology, Psychiatry and Narcology AMS of Ukraine, Vulytsya Akademika Pavlova, 46, Kharkiv 61068, Ukraine. 116. Commission on Ethics Questions of Ternopil Regional Municipal Clinical Psychoneurological Hospital, Vulytsya Troleybusna, 14, Ternopil 46027, Ukraine. 117. West of Scotland Research Ethics Committee 1, Dumbarton Road, Western Infirmary, Glasgow G11 6NT, United Kingdom. 118. Chesapeake Research Review Incorporated Institutional Review Board, 7063 Columbia Gateway Drive, Suite 110, Columbia, MD 21046, United States. 119. Johns Hopkins Medicine Institutional Review Board, 1620 McElderry Street, Reed Hall, Suite B-130, Baltimore, MD 21205-1911, United States. 120. Western IRB, 3535 Seventh Avenue Southwest, Olympia, WA 98502-5010. 121. Saint Joseph's Hospital and Medical Center Institutional Review Board, 350 West Thomas Road, Phoenix, AZ 85013, United States. 122. Mercy Medical Center Institutional Review Committee, 1111 Sixth Avenue, Des Moines, IA 50314, United States 123. Cleveland Clinic Foundation Regulatory Committee, 9500 Euclid Avenue, Desk HSb 103, Cleveland, OH 44195, United States. The Secondary Use Ethics Committee of Biogen gave ethical approval for the use of the ALS samples for this work. Written informed consent to participate in this study was provided by the participants' legal guardian/next of kin.

## Author Contributions

SL and TP: conception or design of the study, data collection, data analysis and interpretation, drafting of the article, and critical revision of the article. CS: contributed to study design and data review. KX: contributed to study design and data analysis. XQ and CG: conception or design of the study, data collection, and data analysis and interpretation. RR: contributed to study design and manuscript development. PC: data analysis and interpretation and critical revision of the article. LS: contributed to assay development by data review and suggested experiments and design of the validation exercises. DG: contributed to data generation, analysis, and interpretation. DR: contributed to data review and interpretation. MM: conception or design of the study, data collection and data analysis and interpretation. AU: conception or design of the study, data analysis and interpretation, drafting of the article, and critical revision of the article. All the authors contributed to the article as described above, and all approved the submitted version.

## Funding

This study was funded by Biogen (Cambridge, MA, United States).

## Conflict of Interest

SL is an employee of Siemens Healthcare Laboratory, LLC. TP, KX, and RR was an employee of Biogen Inc. at the time of the study. TP is currently an employee of Takeda. CS, DG, and DR are employees of Biogen Inc. XQ, CG, and MM was an employee of Siemens Healthcare Laboratory, LLC, at the time of the study. AU is an employee of Siemens Healthcare Laboratory, LLC, has supervised the work of SL, XQ, and MM, and owns shares of Siemens Healthineers AG stocks. Biogen was involved in the writing and editorial support of this article. The remaining authors declare that the research was conducted in the absence of any commercial or financial relationships that could be construed as a potential conflict of interest.

## Publisher's Note

All claims expressed in this article are solely those of the authors and do not necessarily represent those of their affiliated organizations, or those of the publisher, the editors and the reviewers. Any product that may be evaluated in this article, or claim that may be made by its manufacturer, is not guaranteed or endorsed by the publisher.
