# Supplementary material for: Development of a Highly Sensitive Neurofilament Light Chain Assay on an Automated Immunoassay Platform
Source: Front Neurol. 2022 Jul 25;13:935382. doi: 10.3389/fneur.2022.935382 (PMC9359312; doi:10.3389/fneur.2022.935382)
Supplement: Supplementary file 1 [file Data_Sheet_1.docx]

**Supplementary Material**

**Supplementary Figure 1.** Serum sample stability with NfL research assay. Measured NfL concentrations of four individuals (serum) with different handling conditions: (A) room temperature up to 48 hours (B) freeze/thawed up to five cycles. NfL, neurofilament light chain.

**
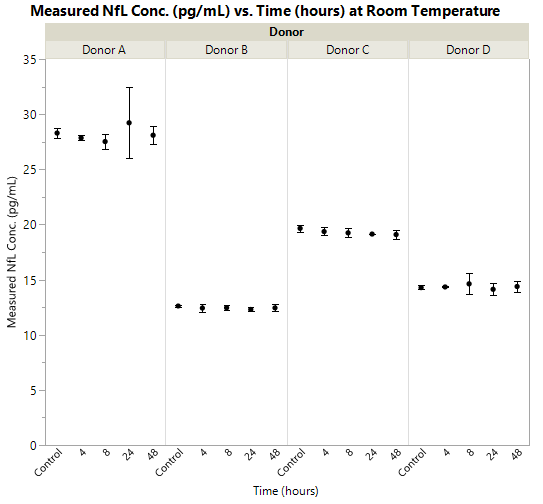
**

**
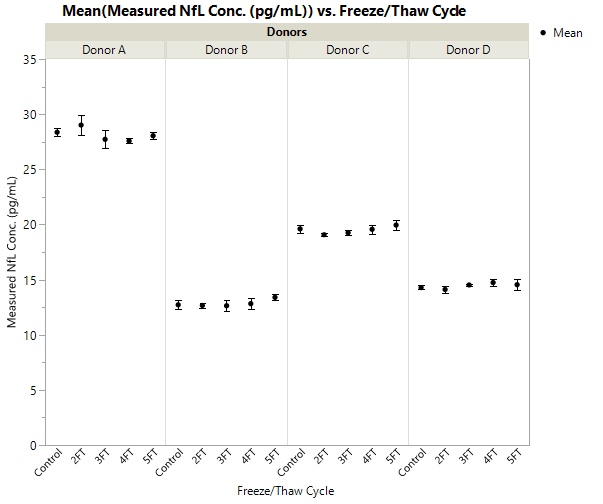
**

**Supplementary Figure 2.** Hook effect evaluation of NfL research assay. No hook effect observed for NfL assay up to 481 ng/mL (~1000-fold of upper limit of quantification). NfL, neurofilament light chain.

**Supplementary Table 1.** Onboard sample stability. Sample is stable through 9 hours of onboard the system with uncapped sample containers stored at 17–25˚C.

| **Test Samples** | | | | | | | | | |
| --- | --- | --- | --- | --- | --- | --- | --- | --- | --- |
|  | **Low NfL Sample** | | | **Medium NfL Sample** | | | **High NfL Sample** | | |
| **Hours** | **Mean** | **% CV** | **% REC** | **Mean** | **% CV** | **% REC** | **Mean** | **% CV** | **% REC** |
| 0 | 7.85 | 2.0% | NA | 49.04 | 1.7% | NA | 369.30 | 2.6% | NA |
| 4 | 8.01 | 1.1% | 102.1% | 48.88 | 3.8% | 99.7% | 363.23 | 4.0% | 98.4% |
| 5 | 8.05 | 6.0% | 102.7% | 48.41 | 4.7% | 98.7% | 353.91 | 1.8% | 95.8% |
| 8 | 7.84 | 3.6% | 99.9% | 46.32 | 3.8% | 94.5% | 360.09 | 5.8% | 97.5% |
| 9 | 8.22 | 1.3% | 104.7% | 46.73 | 1.5% | 95.3% | 351.18 | 2.0% | 95.1% |

%CV = Coefficient of variation. % REC = % Recovery, which is the percentage of the measured NfL concentration to mean NfL concentration at time 0.
